# SpinalTRAQ: A Novel Pipeline for Volumetric Cervical Spinal Cord Analysis Identifies the Corticospinal Tract Synaptic Projectome in Healthy and Post-stroke Mice

**DOI:** 10.1523/ENEURO.0276-25.2025

**Published:** 2025-09-18

**Authors:** Katherine Poinsatte, Matthew Kenwood, Dene Betz, Ariana Nawaby, Apoorva D. Ajay, Wei Xu, Erik J. Plautz, Xiangmei Kong, Julian P. Meeks, Denise M. O. Ramirez, Mark P. Goldberg

**Affiliations:** ^1^Department of Neurology, University of Texas Southwestern Medical Center, Dallas, Texas 75390; ^2^Peter O’Donnell Brain Institute, University of Texas Southwestern Medical Center, Dallas, Texas 75390; ^3^Department of Neurology, Long School of Medicine, University of Texas Health Science Center San Antonio, San Antonio, Texas 78229; ^4^Graduate School of Biomedical Science (Neuroscience), University of Texas Health Science Center San Antonio, San Antonio, Texas 78229; ^5^Department of Neuroscience, University of Texas Southwestern Medical Center, Dallas, Texas 75390; ^6^Departments of Neuroscience, Pediatrics, and Pharmacology & Physiology, University of Rochester, Rochester, New York 14627

**Keywords:** cervical spinal cord, corticospinal tract, motor neuron, plasticity, spinal cord atlas, stroke

## Abstract

The corticospinal tract (CST) is essential for forelimb-specific fine motor skills. In rodents, it undergoes extensive structural remodeling across development, injury, and disease states, with major implications for motor function. A vast body of literature, spanning numerous injury models, frequently assesses these projections. Despite this, a cohesive imaging modality for rapid, quantitative assessment of the bilateral cervical spinal cord projectome is lacking. To address this, we developed SpinalTRAQ (Spinal cord Tomographic Registration and Automated Quantification), a novel mouse cervical spinal cord volumetric reference atlas and machine learning-based analytical pipeline. Using serial two-photon tomography, SpinalTRAQ enables unbiased, region-specific quantification of fluorescently labeled CST presynaptic terminals. In healthy male mice, the CST exhibits a distinct bilateral synaptic projectome, with the densest innervation in laminae 5 and 7 on the contralateral side and lamina 7 on the ipsilateral side. We additionally observed sparse synaptic input in lamina 9, specifically axial motor neuron pools, which we found was targeted to spinal motoneurons. Following focal motor cortical stroke, the injured CST axons are depleted, and contralesional CST projections are significantly increased after 4 and 6 weeks. By 6 weeks post-stroke, ipsilateral CST synapses were increased by fivefold, with the greatest increases seen in homotopic laminae and all motor neuron pools. SpinalTRAQ offers detailed, level- and lamina-specific quantification of the bilateral cervical spinal cord synaptic projectome, revealing previously unrecognized CST connectivity and plasticity after injury.

## Significance Statement

Stroke is a leading cause of disability worldwide, with millions of survivors experiencing long-term motor impairments that severely affect their quality of life. Understanding how the corticospinal tract (CST), a key pathway for motor control, adapts after injury is critical for developing effective rehabilitation strategies. Here, we present SpinalTRAQ, a novel imaging and analysis tool that enables precise, lamina-specific mapping of CST connectivity in the cervical spinal cord. Using SpinalTRAQ, we uncovered significant CST plasticity following stroke, including robust synaptic reinnervation of motor neurons by the uninjured CST. These findings provide new insights into the mechanisms of motor recovery and offer a powerful resource for advancing therapies aimed at enhancing neural repair and restoring motor function in stroke patients.

## Introduction

The corticospinal tract (CST) is the primary descending motor pathway, essential for executing fine motor skills such as reaching and grasping (Heffner and Masterton, 1975; Lemon, 2008; Alstermark and Isa, 2012). In humans, nonhuman primates, and rodents, the CST originates predominantly in the primary motor (M1) and somatosensory cortices, projects through the internal capsule and pyramidal tract, and mostly crosses at the medullary decussation to innervate spinal cord targets (Watson et al., 2021). Despite this shared architecture, CST anatomy differs across species. In primates, CST axons project through lateral and ventral columns and form direct connections with lower motor neurons in lamina 9, enabling highly dexterous movements (Nudo and Masterton, 1990; Lacroix et al., 2004; Lemon, 2019). In rodents, CST axons descend through dorsal columns and mainly innervate interneurons in laminae 4–8 (Ueno et al., 2012; Kaiser et al., 2019), with little direct input to motoneurons (Alstermark and Ogawa, 2004; Lemon, 2008; Gu et al., 2017; Lemon, 2019). While rodents transiently form direct motoneuron connections during development, these connections are later pruned, with a reduction in skilled motor movement (Kamiyama et al., 2006; Gu et al., 2017). The rodent CST fine-tunes its output by precisely targeting premotor interneurons within discrete regions (Bui et al., 2013; Azim et al., 2014; Levine et al., 2014; Ni et al., 2014; Hayashi et al., 2018; Ueno et al., 2018; Ronzano et al., 2022).

The murine CST exhibits remarkable plasticity during development (Martin et al., 2007; Canty and Murphy, 2008; Welniarz et al., 2017), injury (Lapash Daniels et al., 2009; Ueno et al., 2012; Kaiser et al., 2019; Liu et al., 2021; Chen et al., 2022), and disease (Zdunczyk et al., 2018; Lemon, 2021). After unilateral M1 injury, the CST from the damaged hemisphere degenerates, causing significant denervation of the spinal cord that corresponds to a loss of reaching, grasping, and precision motor behaviors (Whishaw et al., 1986; Whishaw et al., 1991; Becker et al., 2016; Becker et al., 2020). In response, CST fibers from the uninjured hemisphere undergo dynamic remodeling, including midline-crossing axons and synaptic reinnervation of denervated regions. The extent of these new projections correlates with recovery of fine motor skills (Liu et al., 2009) and silencing them reinstates deficits (Wiessner et al., 2003; Liu et al., 2013; Bachmann et al., 2014; Wahl et al., 2014). Enhanced CST sprouting in the spinal cord does not consistently lead to behavioral improvement, suggesting that recovery depends on targeted plasticity. The extent and precise location of sprouting needed for functional recovery remain unclear. Thus, while CST sprouting holds significant therapeutic potential, harnessing this phenomenon requires a deeper understanding of its synaptic organization as well as improved tools to map and quantify CST projections within the spinal cord.

Volumetric image analysis workflows provide tools to characterize and rigorously map connectivity in the rodent brain and brainstem (Poinsatte et al., 2019; Wang et al., 2022). Similar approaches are needed to enable robust, scalable quantification in the spinal cord. A key challenge is the standardized registration of volumetric datasets to achieve automated, region-specific quantification. Existing tools serve as pivotal foundations for whole spinal cord analysis (Fiederling et al., 2021a,b), though a reliance on manual sectioning and a limited reference atlas underscores the need for improved pipelines. To address this gap, we developed SpinalTRAQ (Spinal cord Tomographic Registration and Automated Quantification), an integrated imaging and analysis pipeline for region-specific, unbiased quantification of 3D fluorescent images of mouse cervical spinal cords. This pipeline builds on established whole-brain imaging techniques, utilizing custom computing workflows to create an end-to-end pipeline for 3D visualization, atlas registration, machine learning-driven image segmentation, and automated quantification of cervical spinal cord volumetric datasets (Poinsatte et al., 2019; Ramirez et al., 2019; Ortega et al., 2020). Using SpinalTRAQ, we investigated the CST synaptic projectome in healthy and injured adult mice. Specifically, we assessed CST synaptic distribution in the intact spinal cord and tracked changes after focal M1 stroke. This approach builds upon existing characterization of CST axons, which often traverse gray matter without forming synapses, offering a more complete assessment of the CST projectome. Our results reveal substantial reorganization of CST synaptic connectivity, including significant, region-specific reinnervation of denervated areas from the uninjured CST. These data offer critical insights into the mechanisms of CST plasticity and highlight SpinalTRAQ's utility in probing synapse-level changes associated with injury and disease. Ultimately, this platform presents a powerful means to identify and leverage therapeutic targets that promote functional recovery following CNS damage.

## Materials and Methods

### Animals

All animal procedures were performed in accordance with the UT Southwestern or UT Health San Antonio animal care committee's regulations. CST synaptic projectome experiments were performed on 8–11-week-old male C57BL/6 mice (*n* = 30, Jackson Laboratory). ChAT-GFP experiments used 9-week-old male mice (*n* = 3, Jax # 006410 or # 007902) expressing eGFP in cholinergic (ChAT+) neurons. For AAV1-Cre tracing experiments, male 8–16-week-old Sun1-sfGFP (*n* = 3, Jax # 021039) mice were used. Animals were housed in a 12 h light/dark cycle with access to food and water *ad libitum*.

### Photothrombotic stroke

Photothrombotic (PT) stroke was induced in the left motor cortex as described in Yanev et al. (2020). Mice were anesthetized with 1–4% isoflurane in 70% nitrous oxide/30% oxygen and placed in a stereotaxic frame. After a midline scalp incision (aseptic conditions), rose bengal (Sigma-Aldrich; 40 mg/kg, i.p., dissolved in saline) was administered. One minute later, a 45 mW laser (Coherent Sapphire; 561 nm; 2.7 mm collimated beam diameter) targeted 1.7 mm lateral to bregma for 15 min. Sham mice underwent identical procedures without laser exposure. Postoperative care included Buprenorphine ER for analgesia and moist food for 24–72 h. All surgeries were performed successfully by the Neuro-Models Facility at UT Southwestern Medical Center (RRID:SCR_022529).

### Virus production

SynaptoTag4, derived from SynaptoTag2 with an added GAP43-targeted tdTomato, was packaged into an AAVDJ serotype as described (Li et al., 2021). Briefly, AAV-293 cells were cotransfected with pHelper, pRC-DJ, and Synaptotag4 vectors and then harvested after 72 h. Cell lysates underwent 400,000 × *g* centrifugation for 2 h in an iodixanol gradient, and the 40% fraction was washed and concentrated using a 100 kDa cutoff filter. The final viral titer ranged from 0.5–2 × 10^13^ gc/ml (qPCR). Virus was aliquoted and stored at −80°C; SynaptoTag4 was generously provided by Wei Xu's laboratory at UT Southwestern.

### Intracortical AAV injections

For all experiments, mice were anesthetized (1–4% isoflurane in 70% N_2_O/30% O_2_) with breathing rates and temperatures monitored. For CST Synaptic Projectome studies, a burr hole was drilled 1.5 mm lateral to bregma and 1 µl of SynaptoTag4 AAV was pressure-injected into the right motor cortex at –0.6 mm over 10 min, followed by a 10 min wait before pipette removal. Pressure injections were performed using a 2.5 μl Hamilton syringe (Hamilton #7535-01) backfilled with neutral mineral oil and fitted with a pulled glass pipette using an RN Compression Fitting (Hamilton #55750-01). For AAV1-Cre tracing, Sun1-GFP mice received 1 µl pressure injections (Nanoliter2020 Injector, World Precision Instruments) of pAAV-hSyn-Cre-P2A-dTomato (Addgene #107738; 2 × 10^13^, gift from Rylan Larsen) injected (100 nl/min) at –0.2/1.5 (A/P) and –0.5 mm depth into the right motor cortex. The pipette was left in place for 1 min and then raised 0.2 mm, followed by an additional 1 min static hold and then subsequently removed. Postoperatively, mice received Buprenorphine ER and moist food for 24–72 h.

### Sample preparation for imaging on TissueCyte 1000

Two weeks post-injection, mice were perfused with PBS followed by 4% PFA. Tissues were post-fixed in 4% PFA for 24 h at 4°C, and the cervical spinal cord was isolated and stored in 0.01% NaN_3_/PBS until embedding. A 4% SureCast solution (acrylamide:bis-acrylamide, 29:1; Thermo Fisher Scientific #HC2040) containing 0.5% VA-044 (Wako #27776-21-2) was prepared on ice. Cervical spinal cord segments were immersed overnight at 4°C in this mixture and then equilibrated for 1h at room temperature on a shaker. Cervical spinal cord segments were transferred to a disposable mold (VWR #15160-215), covered with 2.5 ml of the acrylamide solution, sealed with aluminum foil, and incubated at 40°C for 2–3 h. In parallel, a 4.5% (w/v) agarose solution (Type 1A, low EEO; Sigma #A0169) in 50 mM phosphate buffer was oxidized with 10 mM NaIO_4_ (Sigma #S1878) and stirred gently for 2–3 h in the dark. The solution was vacuum filtered, washed three times with 50 mM phosphate buffer, and resuspended to a final agarose concentration of 4.5% with PB. Ten milliliters of agarose solution are needed for each spinal cord segment to be embedded. The oxidized agarose solution was heated to boiling in a microwave and then transferred to a stirring plate and allowed to cool to 60–65°C. Spinal cord segments were removed from the oven and molds, and excess agarose was gently removed from the tissues using Kimwipes or tweezers. The cervical segments were embedded by placing a fresh cryoembedding mold (VWR #15560-215) on a flat ice pack, partially filling with 3 ml oxidized agarose heated to 60–65°C, and then quickly submerging the cervical sections using forceps into the bottom of the block with the rostral side touching the bottom of the block (caudal side facing upward). Once the agarose had hardened slightly around the tissue, the rest of the block was filled with agarose until slightly overfilled using a disposable transfer pipette. The agarose block was allowed to fully solidify on a frozen ice pack in the dark. Once the agarose blocks were fully hardened, the tops of the agarose blocks were trimmed with a razor blade until flat and level with the sides of the mold and then the blocks including the specimens were removed from the molds and placed in individual small glass jars (Uline #S-17073M-W), where they were treated overnight at 4°C in the dark in sodium borohydride buffer [50 mM sodium borohydride (Sigma # 452882), 50 mM borax (Sigma, #221732), 50 mM boric acid (Sigma # B6768), pH 9.0–9.5]. After overnight cross-linking, the agarose blocks were transferred to phosphate buffer for storage at 4°C until TissueCyte imaging.

### Serial two-photon tomography

The agarose blocks containing the spinal cord samples were attached to a custom magnetic slide with superglue and placed on a magnetized stage within an imaging chamber filled with phosphate buffer. Serial two-photon tomography (STPT) imaging is a block-face imaging technique in which a series of two-dimensional (2D) mosaic images in the transverse plane are acquired just below the cut surface of the spinal cord, followed by physical sectioning with a built-in vibrating microtome to cut away the imaged tissue, preparing a new cut surface for imaging (Ragan et al., 2012). For this study, three optical planes were imaged at 20, 40, and 60 μm below the cut surface, followed by a vibrating microtome cut at 60 μm (blade vibration frequency of 60 Hz, advancement velocity 0.5 mm/s). The excitation laser (Mai Tai DeepSee, Spectra-Physics/Newport) wavelength was tuned to 780 nm for initial sample positioning and then 930 nm for image acquisition to efficiently excite both GFP and tdTomato present in the cord segments. Spinal cords were positioned for imaging by centering the central canal beneath the objective before moving the stage to the starting position. Three emission channels using pre-set bandpass filters encompassing red, green, and blue emission were collected with a predetermined photomultiplier tube voltage of 720 V. This process produced 57,600 image tiles, 200 physical sections, and 1,800 2D stitched coronal section images (8 by 12 mosaic, 1 stitched coronal section image for each channel and each optical plane) with a lateral resolution of 0.875 μm/pixel and axial resolution of 20 μm (∼280 gigabytes of raw data per cord). The raw image tiles were first trimmed and subjected to flat field correction and then stitched into 2D mosaic coronal section images using the Autostitch software (TissueVision). Single channel, single plane stitched coronal section images were deposited on our cluster computing resource, BioHPC, for further processing and analysis using our custom developed pipelines. TissueCyte imaging was performed in the UT Southwestern Whole Brain Microscopy Facility (RRID: SCR_017949).

### ST-CRA average template creation

The average template was created using averaged autofluorescent signal from the red channel of a set of 20 cervical cord samples imaged on the TissueCyte. The image volumes comprising the average template are available in the Zenodo repository (doi:10.5281/zenodo.14750099). Pre-existing annotations from the SpinalJ atlas were adapted to provide corresponding boundaries for the anatomical regions present in the average template.

Autofluorescence images to be used for the template underwent a preliminary rescaling to better match the space of the SpinalJ annotations. The right hemisphere of these images was mirrored in order to create left–right symmetry between the template hemispheres. The set of images from the 20 cervical cord samples then underwent a process of iterative averaging and re-registration. Images from the 20 samples were linearly averaged into a single preliminary template image. Then each sample was registered into this preliminary template. The registered sample images were then linearly averaged again into a new preliminary template. This process was repeated seven times to create a highly aligned average representation of the underlying autofluorescent anatomy.

To correct remaining misalignment between the average template and the SpinalJ annotations, the aspect ratio of the SpinalJ annotations was manually adjusted to better match the template, by compressing the width to 93% of its original size and compressing the height to 95% of its original size. The template was manually affinely adjusted within each cervical segment to better match the white and gray matter boundaries present in the annotation. The template was then masked using the total area of the annotations, to create clear boundaries between the background and tissue components. A diffuse artificial border was created around the template in order to mimic the higher autofluorescence intensity typically seen at tissue borders with the aim of improving downstream registration accuracy.

### Sample registration

The raw fluorescence signal from the red channel of the stitched 3D images is used to register the dataset to our STPT-based cervical spinal cord average template using a custom pipeline constructed with the SimpleElastix package (Lowekamp et al., 2015). Cord samples were padded with blank section images to 200 sections in the *Z*-dimension if fewer than 200 sections were collected during imaging or cropped to 200 *Z* sections if more than 200 sections were collected, to ensure all cords had the same image dimensions for registration. Because curvature along the length of the spinal cord in the *Z*-dimension can cause the section images to be horizontally and vertically displaced from the center of the atlas template, section images were automatically recentered using a MATLAB script during preprocessing to improve the registration initialization. A multistep registration process was chosen to use rigid, affine, and b-spline registration components. At each step, optimization was performed on a six-resolution multigrid (pyramid) schedule, with Mattes Mutual Information as the optimization metric. Final b-spline grid dimensions were 1 × 1 × 1 mm. In cases where the automated registration alignment was not optimal, an additional registration step was performed, in which the Euclidean distance between manually specified fiducial markers in the sample and atlas was minimized (see below for further details). The optimized registration transformation identified for the raw red channel images was then applied to the other raw image channels.

### Pixel classification and region

Relevant signals of interest were identified in the images using the “Pixel Classification” plugin for the Interactive Learning and Segmentation Toolkit (Ilastik), which implements a random forest supervised machine learning (ML) classifier (Somner et al., 2011). To support the supervised ilastik training, a maximum intensity projection (MIP) was first created for each physical section from its three optical planes. The resultant MIP image was color adjusted to improve fluorescent signal contrast and downsampled to a pixel size of 1.5 μm × 1.5 μm in the *x*–*y* dimension. This resulted in a set of 16 bit three-color compressed TIFF images with the same number of images as physical sections in the original STPT image volume. Up to five representative sections containing examples of fluorescent signals of interest (eGFP+ presynaptic terminals/tdTomato-positive axons or eGFP+ cholinergic neurons) and spanning the rostrocaudal extent of the cervical spinal cord sample were selected from each sample. The random forest ML model was trained on these selected sections by labeling pixels as axons, presynaptic terminals, white matter, gray matter, and other features (noise, background, etc.). After completing the initial training, each of the sample images was iteratively updated until visible misclassifications were minimized. Each cohort of spinal cords (CST synaptic projectome or ChAT-GFP validation experiments) was used to train an independent model for classification, and every section from every spinal cord in the cohort was subjected to the same model. Ilastik predictions were exported as an 8 bit “probability map” TIFF image for each trained label, with the pixel value of 0 mapping to 0% probability and 255 100% probability of a pixel belonging to the label. The resulting TIFF stacks were warped to the custom STPT-based cervical spinal cord average template using the transformation parameters identified during the registration process for the raw fluorescent images. The probability maps were then thresholded (pixel threshold = 86, determined visually) to remove low probability noise. The summed intensity of all denoised probability map voxels lying within annotated regions present in the atlas were quantified using custom MATLAB software, producing a matrix of signal intensity for each sample, label, and cervical spinal cord region. White matter, gay matter, and background pixel classifications, representing different forms of the negative signal of axons and presynaptic terminal pixels, are not quantified and used only for visualization.

### Code accessibility

The code/software described in the paper is freely available online doi:10.5281/zenodo.14750099 and doi:10.5281/zenodo.13367689.

### Manual fiduciary point placement

In cases where raw image autofluorescence differed substantially from the average template, manual fiduciary point placement was used to fix misaligned images within individual animals. The average template and autofluorescence channel were both opened in ImageJ, and the point tool was used to add points on both image sets that correspond to tissue architecture in both image sets. Routinely marked regions include central canal, gray/white matter boundary with the dorsal funiculus at the midline, gray/white matter boundary of the ventral funiculus at the midline, lateral and medial edges of the dorsal horns, ventral most point of the gray/white matter boundary, and substantially complex ventral horn gray/white matter boundary of lower cervical level such as the forearm motor pool. On average 50–100 points are selected in each sample. A custom ImageJ script was used to obtain coordinates of both images with the point indices.

### Immunofluorescence

For validation of corticomotoneuronal synapses in AAV1-Cre injected mice, mice were transcardially perfused with 1× PBS followed by 4% PFA. Cervical spinal cords were dissected and fixed overnight in 4% PFA, followed by two nights of incubation in 30% sucrose in 1× PBS (w/v). Spinal cords were sectioned via microtome at 45 µm, photobleached utilizing a X-cite120 metal halide lamp and a Quad transmission/excitation filter (Chroma: 89401: 405/488/555/640nm) for 5 min, and stained with anti-ChAT primary antibodies (EMD MilliporeSigma:AB144P, 1:200; Thermo Fisher Scientific: CL3173 1:200) and anti-GFP (Thermo Fisher Scientific: G10362, 1:200) with secondary antibody donkey anti-goat Alexa488 (Thermo Fisher Scientific: A11055; 1:500) and donkey anti-rabbit Alexa564 (Thermo Fisher Scientific: A10042; 1:500) or goat anti-mouse Alexa647 (Thermo Fisher Scientific: A21236; 1:500). Sections were mounted and coverslipped, and images were acquired using a Zeiss LSM710 confocal microscope at 25× magnification.

### Assessment of viral labeling efficiency

Thoracic and lumbar spinal cords were collected from Synaptotag-injected mice, stored for 24 h in 4% PFA and then cryoprotected in 30% sucrose for a minimum of two nights. Thoracic and lumbar spinal cords were embedded in OCT and cryosectioned at 30–40 µm. After DAPI staining (1:5,000), sections were mounted and coverslipped and whole slide images were acquired on the Zeiss Axioscan.Z1 in the UT Southwestern Whole Brain Microscopy Facility (RRID:SCR_017949). All brains were additionally imaged on the TissueCyte following standard published protocols (Poinsatte et al., 2019; Ramirez et al., 2019). Images (data not shown) were assessed to determine sufficient stroke induction and if sufficient viral labeling of the CST was achieved. Confirmation of PT stroke and robust CST labeling was observed in 21 animals, and their cervical spinal cords were processed for STPT and SpinalTRAQ analysis. Nine animals were excluded because of either insufficient intracortical M1 labeling coverage via TissueCyte imaging or poor thoracic CST labeling.

### Experimental design and statistical analysis

CST projectome studies—8–11+week-old male C57BL/6 mice (Jackson Laboratory, *n* = 30) received motor cortex injections of SynaptoTag4. Mice were assigned to four groups—Sham (*n* = 4), 1 week post-stroke (1WPS; *n* = 4), 4 weeks post-stroke (4WPS; *n* = 5), and 6 weeks post-stroke (6WPS; *n* = 4). SynaptoTag4 was injected 2 weeks prior to the experimental endpoint;, thus the 1WPS was injected 1 week pre-stroke, 4WPS at 2 weeks post-stroke, and 6WPS at 4 weeks post-stroke. Every brain and spinal cord were processed for STPT, and 17 mice were retained for final analyses after verification of stroke induction and robust viral labeling. ChAT-GFP experiments—9-week-old male ChAT reporter mice (*n* = 3, Jackson Laboratory #007902)—were processed identically to other studies. AAV1-Cre Tracing experiments—8–16-week-old male LSL-1Sun1-sfGFP mice (*n* = 3, Jackson Laboratory #021039)—were injected with pAAV1-hSyn-Cre-P2A-dTomato and sacked 4 weeks after injection to confirm cortico-motoneuron synapses.

Heatmaps and overlayed dendrogram clustering were analyzed using ComplexHeatmap package (version 2.10.0). Statistical comparisons between cervical levels were compared using a Kruskal–Wallis with subsequent Dunn’s test for multiple comparisons with false discovery rate test correction. *p* values < 0.05 were considered significant; *p* values < 0.07 are reported on graph. Log_10_FC was calculated using classified synapse signal of Group A divided by Group B depending on stated comparison. All data were previously Log_10_ scaled. Data were visualized and graphs were generated using custom R scripts, syGlass (syGlass 1.8), and in Prism (GraphPad Prism 6.0). syGlass volumetric objects were created using ∼200 raw fluorescent MIP images or ilastik pixel probability maps overlayed with atlas regions. Varying *Z*-voxel sizes were used to create pseudo-MIP across multiple images utilizing the cut tool to isolate registered individual cervical levels.

## Results

### SpinalTRAQ: spinal cord tomographic registration and automatic quantification pipeline for region-specific quantification of the cervical spinal cord

**Figure 1.**
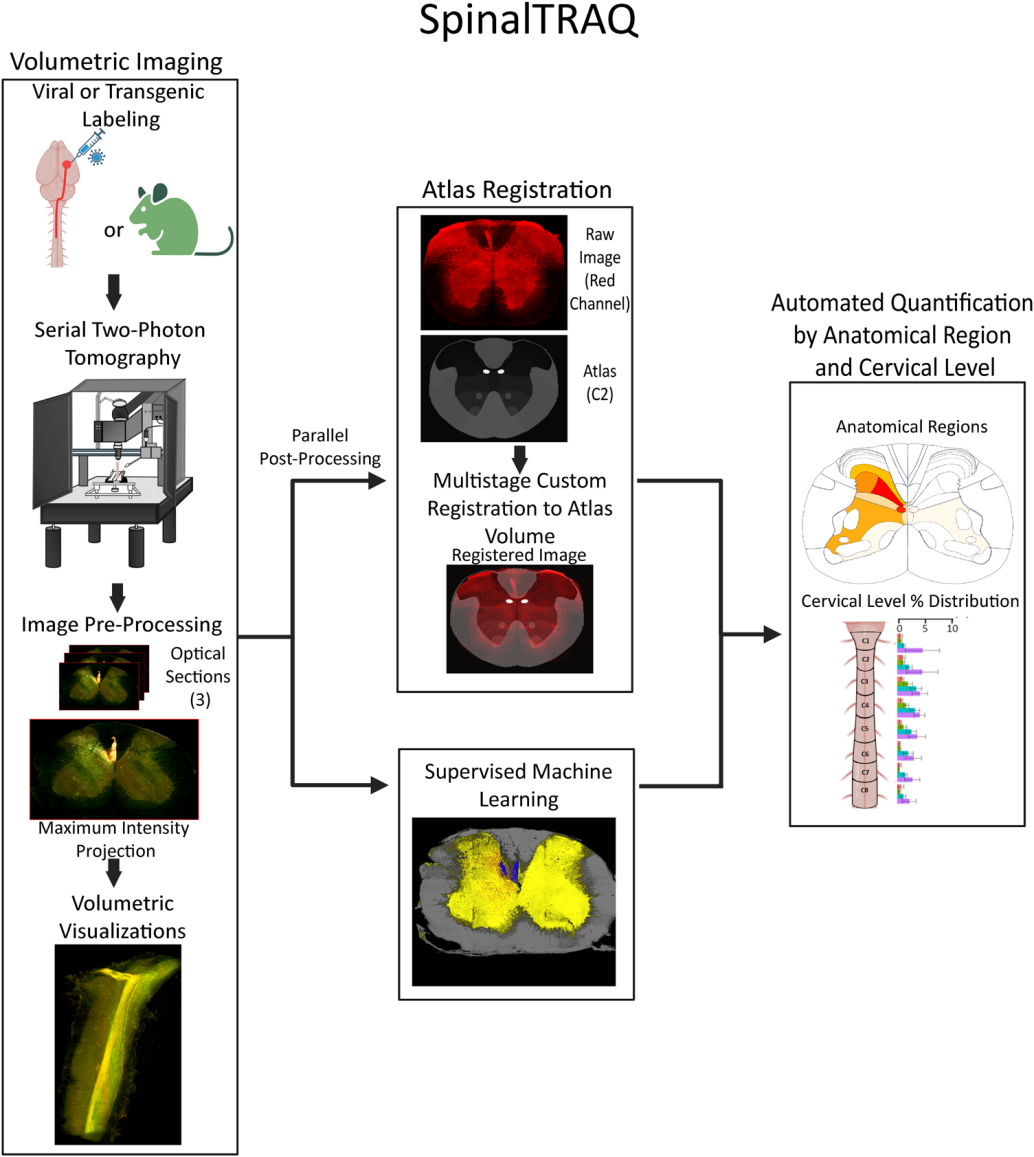
SpinalTRAQ: application of a digitally annotated spinal cord atlas and custom automated pipeline for unbiased region-specific quantification in volumetric image stacks of mouse cervical spinal cord acquired via STPT. Graphical depiction of SpinalTRAQ workflow. Left, Mouse spinal cord is fluorescently labeled using viral or transgenic methods and imaged using STPT (TissueCyte 1000). Images are preprocessed and visualized in 3D. Middle, Parallel computational workflows allow automated registration of cervical cord volumes into a digital atlas and pixel classification of relevant fluorescent signals via supervised machine learning. Right, Registration of pixel classified images (i.e., probability maps) into the reference atlas and custom scripts are used to generate quantitative results on the location of presynaptic terminals or other fluorescent signals of interest across cervical cord laminae and levels.

The SpinalTRAQ pipeline consists of (1) volumetric serial two-photon tomography (STPT) imaging of fluorescently labeled cervical spinal cords; (2) image processing including (a) registration of STPT images into a custom cervical spinal cord atlas and (b) supervised machine learning-driven pixel classification of fluorescent signals; and (3) automated region-specific quantification of classified pixels or objects (Fig. 1).

**Figure 2.**
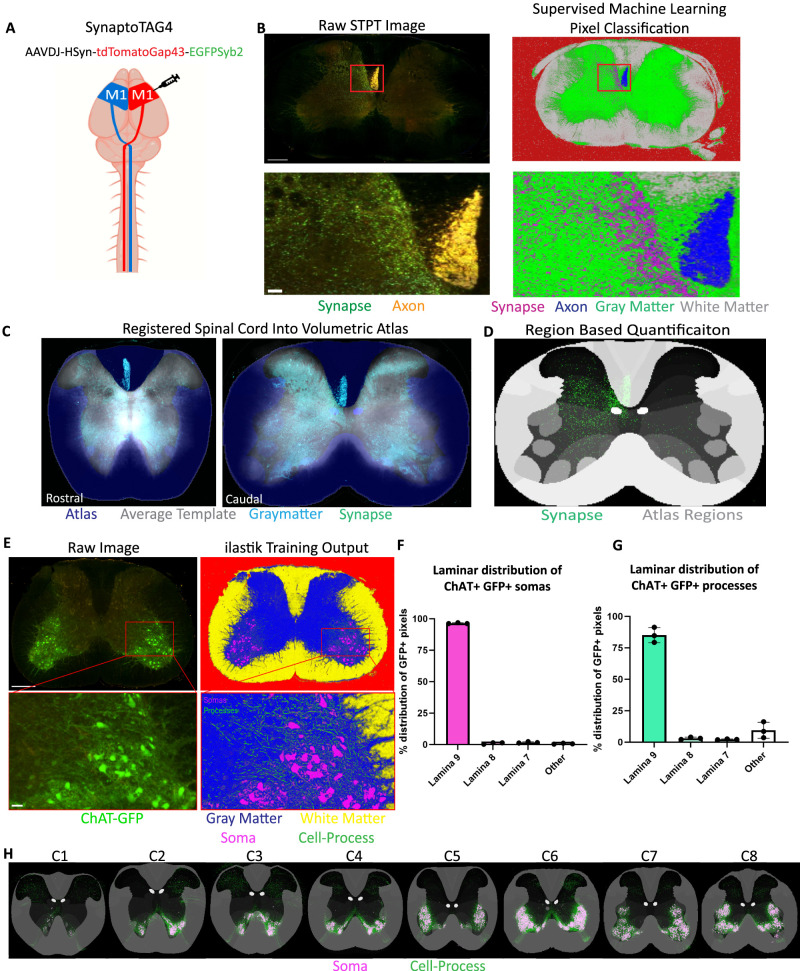
Classifying the CST synaptic projectome in the whole mouse cervical spinal cord. ***A***, Graphical representation of experimental design, showing a unilateral motor cortex injection of the AAV SynaptoTag4 (AAVDJ-HSyn-tdTomatoGap43-EGFPSyb2-WPRE) into healthy adult mice. ***B***, Maximum intensity projection of three full-resolution raw optical planes of the entire cord section (top left panel; scale bar, 400 µm) with zoomed-in section of laminae 4/5 (bottom left panel showing red boxed area; sclae bar, 50 mm). GFP+ presynaptic terminals are shown in green and dual GFP and tdTomato-positive axons residing in the CST are shown in yellow. The right panels show the output of supervised machine learning classification via Ilastik, identifying presynaptic terminals/synapses (magenta), axons (blue), gray matter (green), white matter (gray), and background (red) in the same image. ***C***, Spinal cord autofluorescence (cyan) registered into the average template (gray) at C3(left) and C7 (right). ***D***, Representative example of a single image's positive GFP pixel probability map (green) in digital space with the region annotation overlay (gray). ***E***, Representative images of a spinal cord section from a transgenic ChAT-GFP reporter mouse. Left, Raw fluorescent images of a whole section (top; scale bar, 400 mm) and enlarged area showing ventral horn region (bottom; scale bar, 50 mm) with GFP+ motoneurons. Corresponding pixel classified images (probability maps) after training to identify motoneuron somas (magenta), dendritic processes (green), white matter (yellow), gray matter (blue), and background (red) are shown at right. ***F***, Quantification of the percentage of ChAT+ GFP+ somas and (***G***) dendritic processes found within the lamina 9 motor pools delineated by the digital atlas. ***H***, Condensed probability maps showing somatic (magenta) and neuronal process (green) labeling in one representative ChAT-GFP animal for the indicated cervical levels C1 through C8.

We established our pipeline by mapping corticospinal tract (CST) axons and presynaptic terminals in the cervical spinal cord of adult male C57BL/6J mice. The CST was labeled with SynaptoTag4, a custom adeno-associated virus that drives eGFP expression in presynaptic boutons and tdTomato in descending axons (Fig. 2*A*,*B*; Extended Data Fig. 2-1; Li et al., 2021). Volumetric cervical spinal cord datasets were acquired by STPT imaging (Fig. 2*B*, left panels; Movies 1, 2) at three optical depths. Next, raw fluorescent signal was converted to classified signals of interest using supervised machine learning-based pixel classification via ilastik, which generates probability maps—images in which each pixel's brightness reflects its likelihood of belonging to the target class—and can be thresholded to retain the final classified signals. GFP-positive synapses, tdTomato-positive axons, gray matter, white matter, and background of the image were clearly and accurately segmented (Fig. 2*B*, right panels).

10.1523/ENEURO.0276-25.2025.f2-1 Figure 2-1 **Whole brain SynaptoTag4 STPT imaging** (A) STPT subset of whole brain imaging of SynaptoTag4 inject healthy adult mouse with images spaced 975μm apart from each subsequent image. Download Figure 2-1, TIF file.

**Movie 1.** STPT reconstructed rostral to caudal flythrough of a mouse cervical cord. [View online]

**Movie 2.** STPT reconstructed rotational video of a mouse cervical cord imaged. [View online]

**Movie 3.** Flip-through video of the ST-CRA. [View online]

STPT yields high-resolution image volumes that must be registered to a true volumetric atlas to increase anatomical precision and generalizability across animals for quantification. A current spinal cord atlas tool, SpinalJ (Fiederling et al., 2021a,b), provides a useful framework for conventional light microscopy of traditional histological sections. However, the annotation is derived from select 2D sections of a single animal with spacing reaching >1 mm between reference sections, overlooking the intrinsic complexity of spinal cord regions and inter-animal variability. To address this, we generated a high-resolution 3D template by averaging 620 STPT sections (20 μm spacing) across 20 male C57BL/6J mice. This 3D template was then spatially registered and manually aligned into digital anatomical annotations of C1–C8 published in the SpinalJ package (Fiederling et al., 2021a,b), to create the SpinalTRAQ Cervical Spinal Cord Common Reference Atlas (ST-CRA), analogous to the Allen Institute Mouse Brain Common Coordinate Framework V3 (Fig. 2*C*,*D*). Cervical cord image volumes, including probability maps, were registered into the ST-CRA for region-specific quantification (Fig. 2*D*, Movie 3). Quantification of classified signal pixel density within 94 volumetric regions defined between C1 and C8 was performed using custom MATLAB scripts. Each step of this pipeline, including pixel classification, ST-CRA registration, and region-specific quantification, was manually inspected for accuracy and validity (data not shown).

Lastly, we tested the accuracy of SpinalTRAQ’s region-specific quantification using transgenic mice expressing GFP under the choline acetyltransferase (ChAT) promoter (Fig. 2*E*). ChAT expression is limited to spinal motoneurons in lamina 9 and sparse interneuron populations (Shneider et al., 2009; Gotts et al., 2016). Comparison of the probability map images (Fig. 2*E*, right panels) with the original raw fluorescent images (Fig. 2*E*, left panels) indicates accurate classification of fluorescent pixels as gray matter, white matter, GFP+ soma, or GFP+ processes. Then, 96.43 ± 0.2% of pixels classified as ChAT GFP+ somas were located within lamina 9 (*n* = 3; Fig. 2*F–H*). A small proportion of classified soma GFP+ pixels were localized to laminae 7 (1.77 ± 0.3%) and 8 (1.08 ± 0.38%), neighboring regions to lamina 9 (Fig. 2*F–H*).

### The level- and lamina-specific CST spinal synaptic projectome in healthy, adult mice

**Figure 3.**
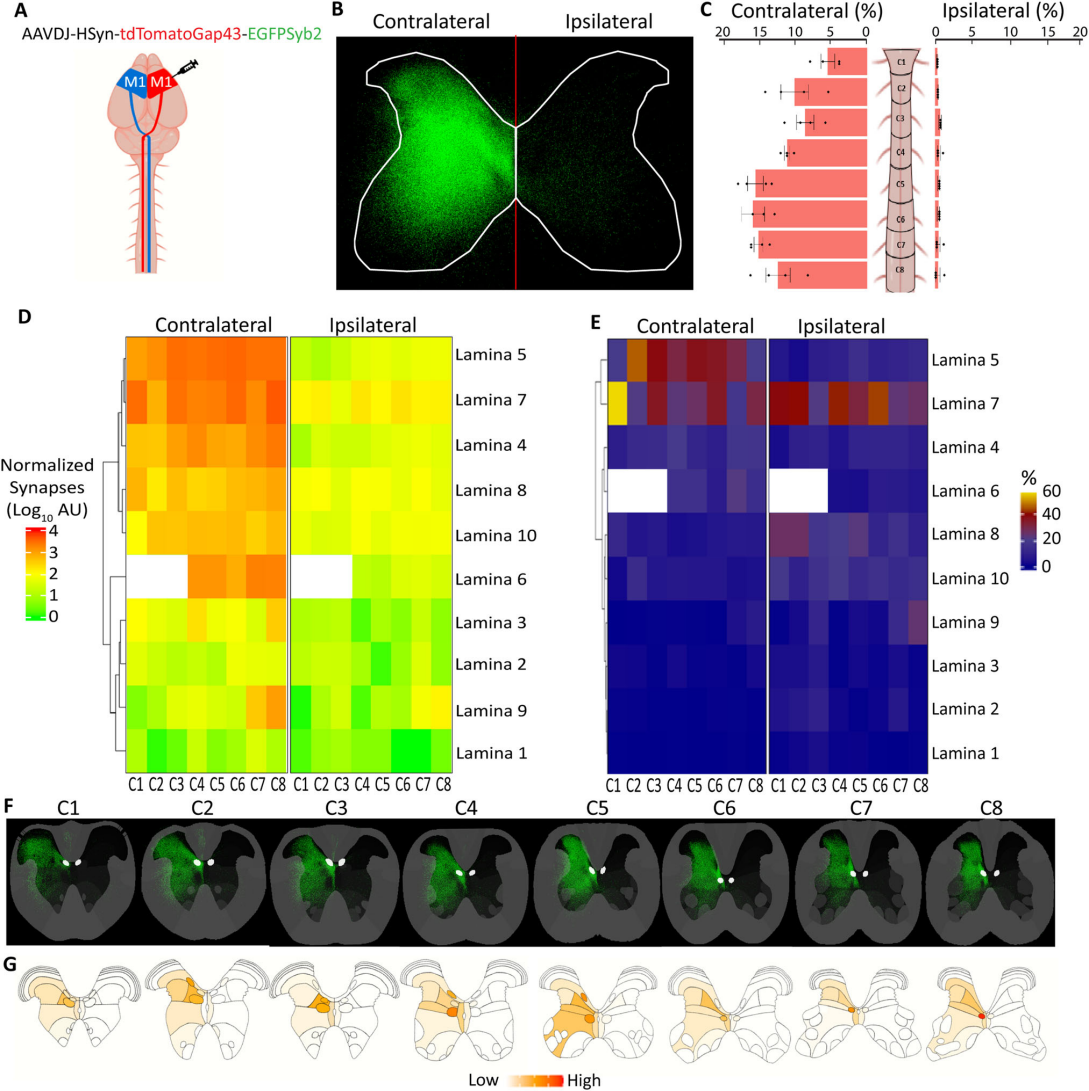
Region- and lamina-specific quantification of the cervical CST synaptic projectome in healthy adult mice. ***A***, Graphical representation of experimental design, showing a unilateral motor cortex injection of the AAV SynaptoTag4. ***B***, Combined probability maps of classified presynaptic terminal pixels across all images in the volumetric spinal cord dataset of a representative animal condensed and shown as a flattened image of the 3D volume. ***C***, Distribution of presynaptic terminals in each cervical level (C1–C8) of both the contralateral and ipsilateral hemicord, shown as the proportion (±SEM) of the total presynaptic terminals for each individual mouse present only in the gray matter. ***D***, Heatmap of presynaptic terminals showing the group mean of normalized synapses on a log10 scale at each cervical level and lamina. Laminae are organized according to dendrogram clustering based on presynaptic terminals in the contralateral hemicord. Empty boxes reflect that lamina 6 is not present at levels C1–C3. ***E***, Distinct laminar distributions of presynaptic terminals in contra- and ipsilateral cords. Values represent relative percentage of the laminae for each cervical level (=100, vertical). ***F***, Presynaptic terminal probability maps for each cervical level (C1–C8) condensed and shown as flattened images of the 3D volumes. ***G***, Graphical depiction of mean synapse distribution in gray matter subregions (*n* = 4).

CST axons were primarily visualized in the cervical hemicord contralateral to brain tracer injection, in the dorsal column and dorsolateral funiculus. Interestingly, in some animals, we also observed sparse labeling in the ipsilateral dorsal column and ipsilateral ventrolateral funiculus, suggesting that pruning of developmental CST pathways, the dorsolateral and ventral CST (dlCST and vCST), may be highly variable between animals (data not shown). Globally, the distribution of CST presynaptic terminals supported prior axon-tracing results, with dense labeling in the contralateral gray matter (96.0 ± 1.2% of labeled synapses; *n* = 4 mice) and a smaller but measurable fraction on the ipsilateral side (3.96 ± 1.26%, *n* = 4 mice; Fig. 3*A–C*; Movies 4, 5). On the segmental level, both hemicords followed a comparable rostrocaudal pattern of CST innervation, with increased synaptic signal found in more caudal sections. Contralateral segments C1–C8 individually contained 5.6–16.1% of the total CST synapse signal for that hemicord, and ipsilateral segments ranged from 0.30% to0.71% (Fig. 3*C*).

10.1523/ENEURO.0276-25.2025.f3-1 Figure 3-1 C**S**T **Synapses in traditional white matter regions and ventral horn.** (A) (Left) eGFP + presynaptic terminals in raw fluorescent image of a healthy adult mouse spinal cord section (scale bar 400μm). (Right) Zoomed inset of left image of eGFP + presynaptic terminals in gray-matter dendritic arbors into lateral funiculus (scale bar 50μm). (B) eGFP + presynaptic terminals in zoomed ventral horn raw fluorescent image of a healthy adult mouse spinal cord section from C1-C8 with Allen Spinal Cord Atlas lamina 9 inset (scale bar 50μm). Download Figure 3-1, TIF file.

**Movie 4.** Mouse cervical cord registered into SpinalTRAQ average template/volume with classified synapses. [View online]

**Movie 5.** Rotational video of a mouse cervical cord with classified synapses. [View online]

At the laminar level, the ipsilateral and contralateral innervation patterns diverged. Contralaterally, laminae 5 and 7 were the most densely innervated at most cervical levels (Fig. 3*D*), containing up to 60% of signal at C1, at least 30% at C2–C6, and at least 20% at C7 and C8 (Fig. 3*E*). Laminae 4, 6, 8, and 10 were highly targeted regions and, as expected, laminae 1–3 had very sparse signal (Fig. 3*D*). Additionally, hierarchical clustering grouped laminae 5 and 7 and laminae 8 and 10, indicating highly similar terminal distributions throughout most cervical levels (Fig. 3*E*; Ueno et al., 2018). On the ipsilateral side, lamina 7 remained a highly targeted region, containing 20–40% of signal at most cervical levels, though lamina 5 was not, containing 2.6–14.5% of total synaptic signal versus 18.9–45.9% on the contralateral side (Fig. 3*E*). Furthermore, the more medial laminae 8 and 10 had the next highest terminal densities in the ipsilateral hemicord, rather than 4 and 6 (Fig. 3*D*,*F*,*G*).

**Figure 4.**
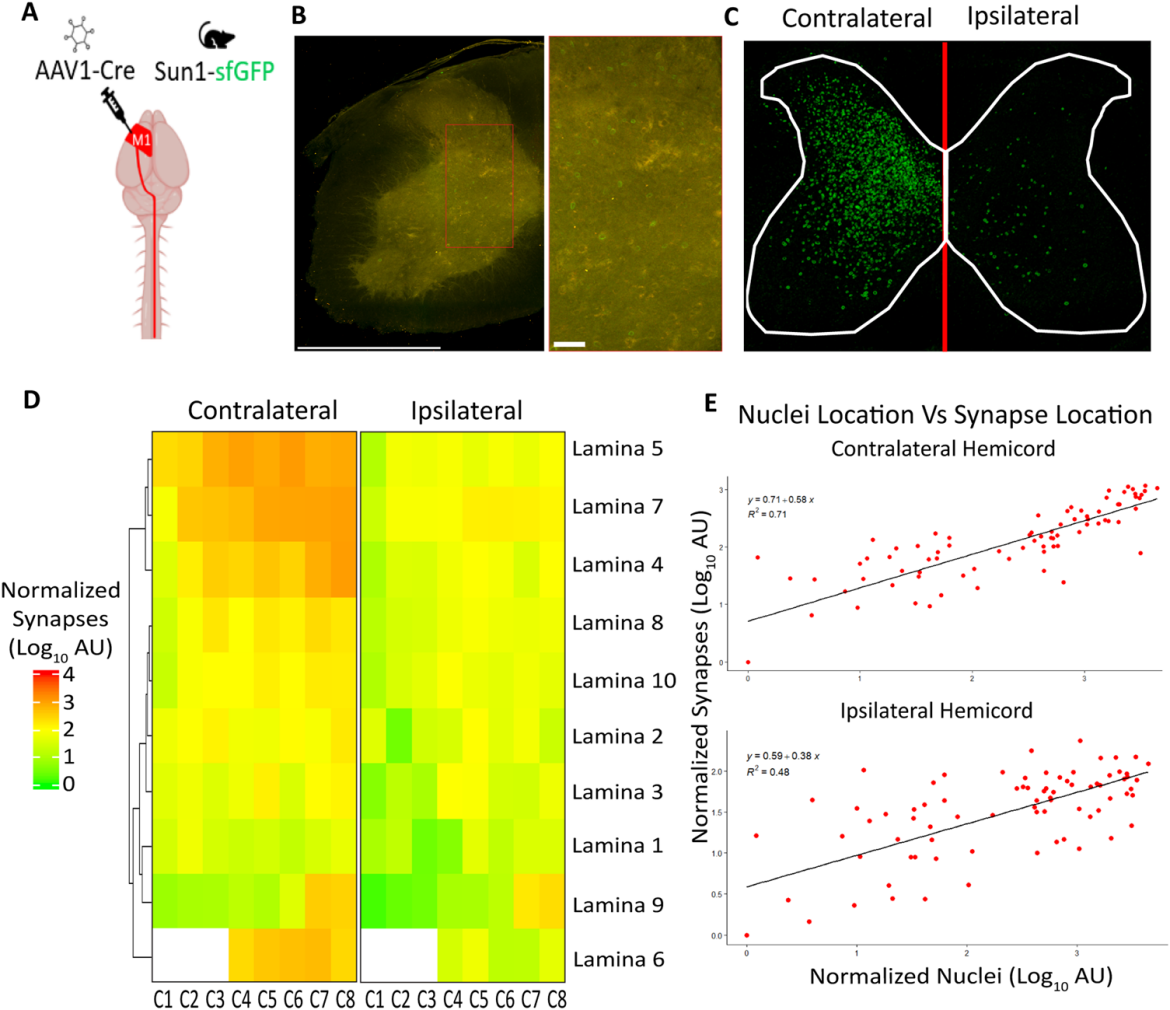
Region- and lamina-specific quantification of the cervical CST secondary neuronal targets using AAV1 in healthy adult mice. ***A***, Graphical representation of experimental design, showing a unilateral motor cortex injection of the AAV1-cre into Sun1-sfGFP mice. ***B***, MIP of five cervical spinal cord sections of a representative Sun1-sfGFP animal showing the contralateral hemicord after imaging on the TissueCyte 1000. ***C***, MIP of probability maps of classified sfGFP nuclear pixels across all images in the volumetric spinal cord dataset of a representative animal. ***D***, Heatmap of classified pixels showing the group mean of normalized nuclear signal on a log10 scale at each cervical level and lamina. Lamina are organized according to dendrogram clustering based on presynaptic terminals in the contralateral hemicord. Empty boxes reflect that lamina 6 is not present at levels C1–C3. ***E***, Scatterplot of SpinalTRAQ gray matter log10 mean normalized synapses (Fig. 3*D*) and log10 mean normalized nuclei pixels (Fig. 4*D*) with a linear best fit line for correlation (*n* = 3).

We mapped the location of CST synaptic targets using a monosynaptic viral tracer, high-titer AAV1-hSYN-Cre-P2A-dTomato in CAG-LSL-SUN1/sfGFP mice (Mo et al., 2015; Carmona et al., 2024) to relate synapse location to postsynaptic target cell bodies. This viral vector exhibits trans-synaptic spread only to first-order connections, inducing Cre-dependent sfGFP expression in the nuclear membranes of postsynaptic CST targets (Fig. 4*A*,*B*; Zingg et al., 2017; Zingg et al., 2020). The distribution of neuronal nuclei was highly correlated with synapse density in the contralateral hemicord (*R*^2^ = 0.71) and moderately correlated in the ipsilateral hemicord (*R*^2^ = 0.48), supporting the specificity and accuracy of AAV1-Cre for monosynaptic labeling of CST spinal targets (Fig. 4*D*,*E*).

### The CST directly innervates lamina 9 in healthy adult mice

**Figure 5.**
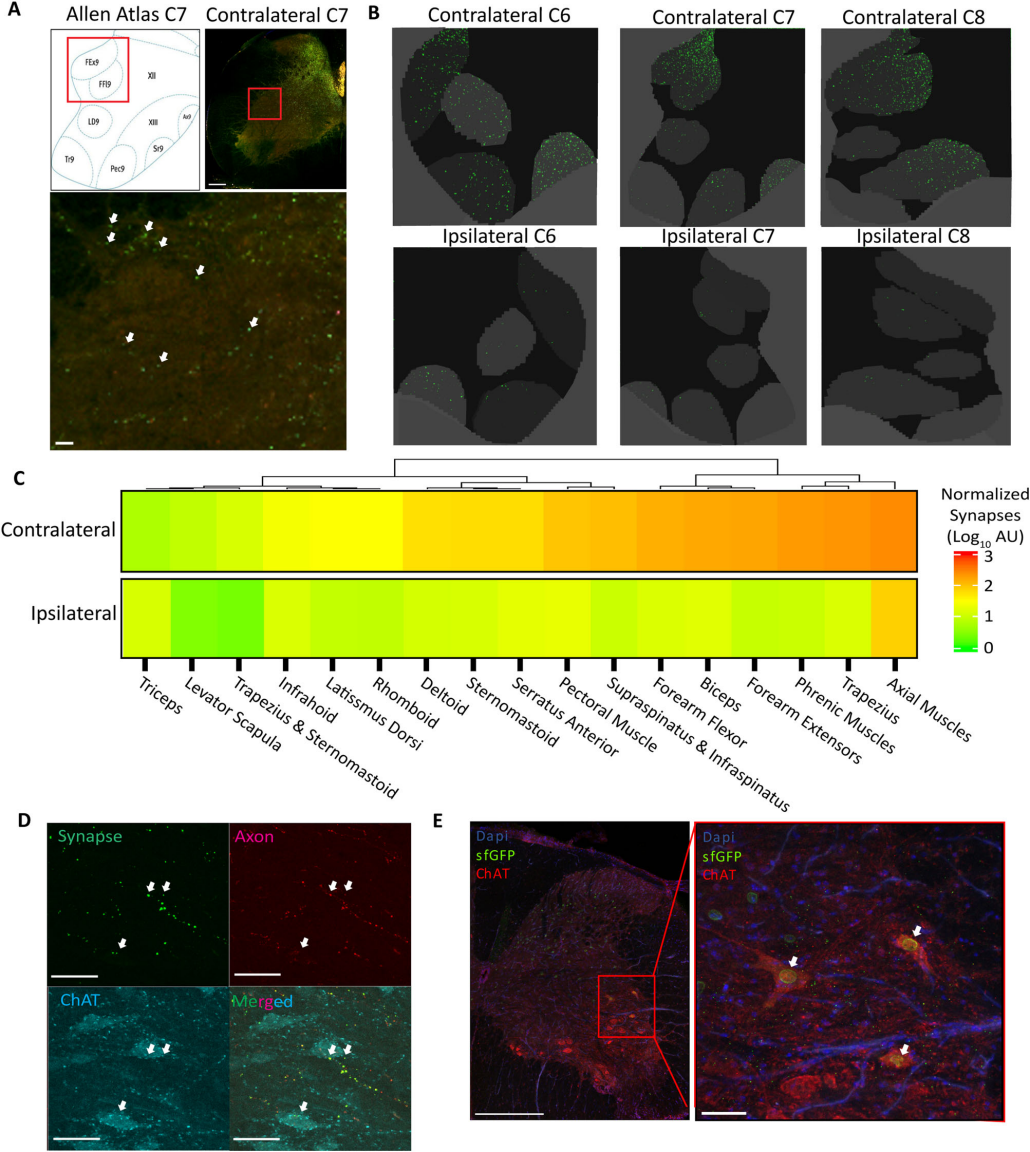
Direct CST synaptic connections with ChAT+ motoneurons in healthy adult mice. ***A***, eGFP + presynaptic terminals in raw fluorescent image of a healthy adult mouse spinal cord section (scale bar, 400 mm) with zoomed-in image of the ventral horn (red boxed area in top right panel; scale bar, 10 mm). White arrows denote presynaptic terminals. Top left panel shows corresponding area of the atlas. ***B***, Cervical levels C6–C8 from the same animal in both hemicords showing condensed presynaptic terminal probability maps (green), masked to show only the ventral motoneuron pools (gray). ***C***, Quantification of mean presynaptic terminal levels for each motoneuron pool throughout the cervical cord across both hemicords, clustered using a dendrogram for the contralateral side (*N* = 4). ***D***, Colocalization of synaptic terminals and ChAT+ motor neurons. Representative confocal *z*-stack of the contralateral ventral horn of a Synaptotag4-injected mouse immunostained with anti-ChAT antibodies. Synaptic GFP is shown in green, axonal tdTomato in red, and ChAT immunolabeling in blue (scale bar, 50 mm). ***E***, Confocal images of a spinal cord section from an AAV1-injected Sun1-eGFP mouse, showing sfGFP+ nuclei (green) within ChAT+ motoneuron cell bodies (red) in a motoneuron pool within the contralateral hemicord. Left, Whole hemicord image (scale bar, 400 mm). Right, Enlarged area boxed in red of a motoneuron pool (scale bar, 50 µm).

Although the CST is known to target lamina 9 in primates, the existence and extent of such connections in mice remain contested (D'Acunzo et al., 2014; Fait et al., 2024). In our studies of healthy adult mice, we found bilateral innervation of lamina 9, ranging from 0.06 to 7.3% of all CST signal at a given cervical level (Fig. 3*E*). Manual assessment of raw fluorescent images confirmed eGFP + presynaptic terminals within or around motoneuron pools in every animal used in this study (Fig. 5*A*, Sup. 1*B*). Maximum intensity projections of classified CST terminals overlaid onto motoneuron pools underscore the prevalence of these connections in both hemicords (Fig. 5*B*). Overall synaptic density was higher in the contralateral cord, where it localized to C7 and C8 (Fig. 3*D*). Ipsilateral lamina 9 projections also primarily targeted C7 and C8, and, interestingly, comprised a greater proportion of ipsilateral CST projections compared with the contralateral hemicord. In fact, laminae 7 and 9 received nearly the same degree of innervation at C8, with 25.7% of total ipsilateral CST projections in lamina 7 and 23.8% in lamina 9 (Fig. 3*E*).

Next, we evaluated the pattern of CST innervation across distinct motoneuron pools, given their precise control of innervated musculature (Stifani, 2014). Contralateral CST projections targeted most motoneuron pools, including the trapezius, phrenic muscle, forearm extensor, biceps, and forearm flexor (Fig. 5*C*). In contrast, the axial muscle motoneuron pool was the only region with significant bilateral innervation.

We next evaluated whether motoneurons could be postsynaptic partners of these lamina 9 synapses. Spinal cord sections from SynaptoTag4-injected mice were immunostained with anti-ChAT antibodies, revealing CST presynaptic terminals in close apposition with ChAT+ motoneurons (*N* = 3 mice; Fig. 5*D*). We further validated this finding by labeling monosynaptic partners, using the AAV1-induced sfGFP+ nuclei paradigm described earlier (*n* = 3). Colocalization of ChAT immunoreactivity with AAV1-induced sfGFP+ nuclei was observed in lamina 9, providing convergent evidence that ChAT+ motoneurons receive direct synaptic input from the CST in the healthy, adult mouse (Fig. 5*E*).

### The post-stroke dynamics of the uninjured CST synaptic projectome

**Figure 6.**
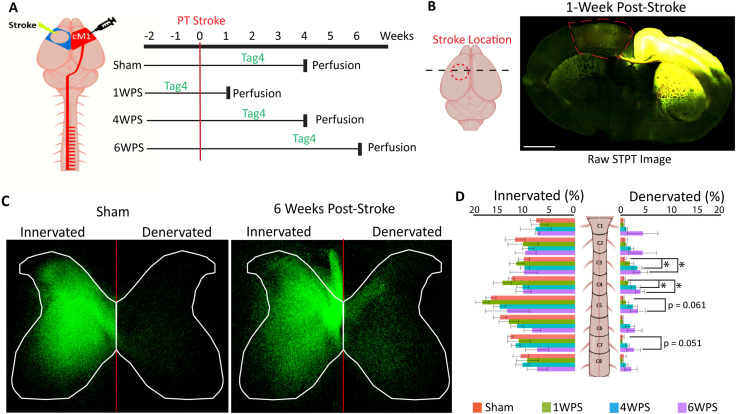
Post-stroke contralesional CST synaptic reinnervation in the denervated cervical hemicord. ***A***, Graphical diagram depicting experimental setup for photothrombotic (PT) stroke and injection of Synaptotag virus to contralateral motor cortex (scale bar, 1 mm). Mice were killed at 1, 4, or 6 weeks post-stroke (WPS). ***B***, Representative coronal image of stroke (traced in red) and virus injection area (yellow) at 1 week post-stroke imaged via STPT. ***C***, Comparison of condensed presynaptic terminal probability maps (C1–C8) from a sham and 6 week post-stroke animal with similar prenormalization viral labeling. ***D***, Distributions of CST presynaptic terminals at each cervical level (C1–C8) between both gray matter hemicords across all four groups, where each mouse's entire cervical spinal cord totals 100%, error bars are SEM (Sham: *n* = 4, 1 WPS: *n* = 4, 4 WPS: *n* = 5, 6 WPS: *n* = 4).

Recovery from M1 injury is associated with robust neuroplasticity in numerous pathways, including the uninjured CST (Lapash Daniels et al., 2009; Liu et al., 2013; Carmichael et al., 2017; Liu et al., 2021; Chen et al., 2022). While increased midline-crossing axons of the uninjured CST have been observed in stroke-denervated spinal regions, very little is known about synaptogenesis by these sprouting axon collaterals, including the time course and precise anatomical location (Liu et al., 2013; Kaiser et al., 2019). We used SpinalTRAQ to assess the dynamic response of the uninjured CST spinal synaptic projectome to stroke, providing region-specific quantifications of spontaneous neuroplasticity during the window for stroke recovery. The uninjured CST was labeled at three key recovery time points: 1, 4, and 6 weeks post-stroke (Fig. 6*A*,*B*).

**Figure 7.**
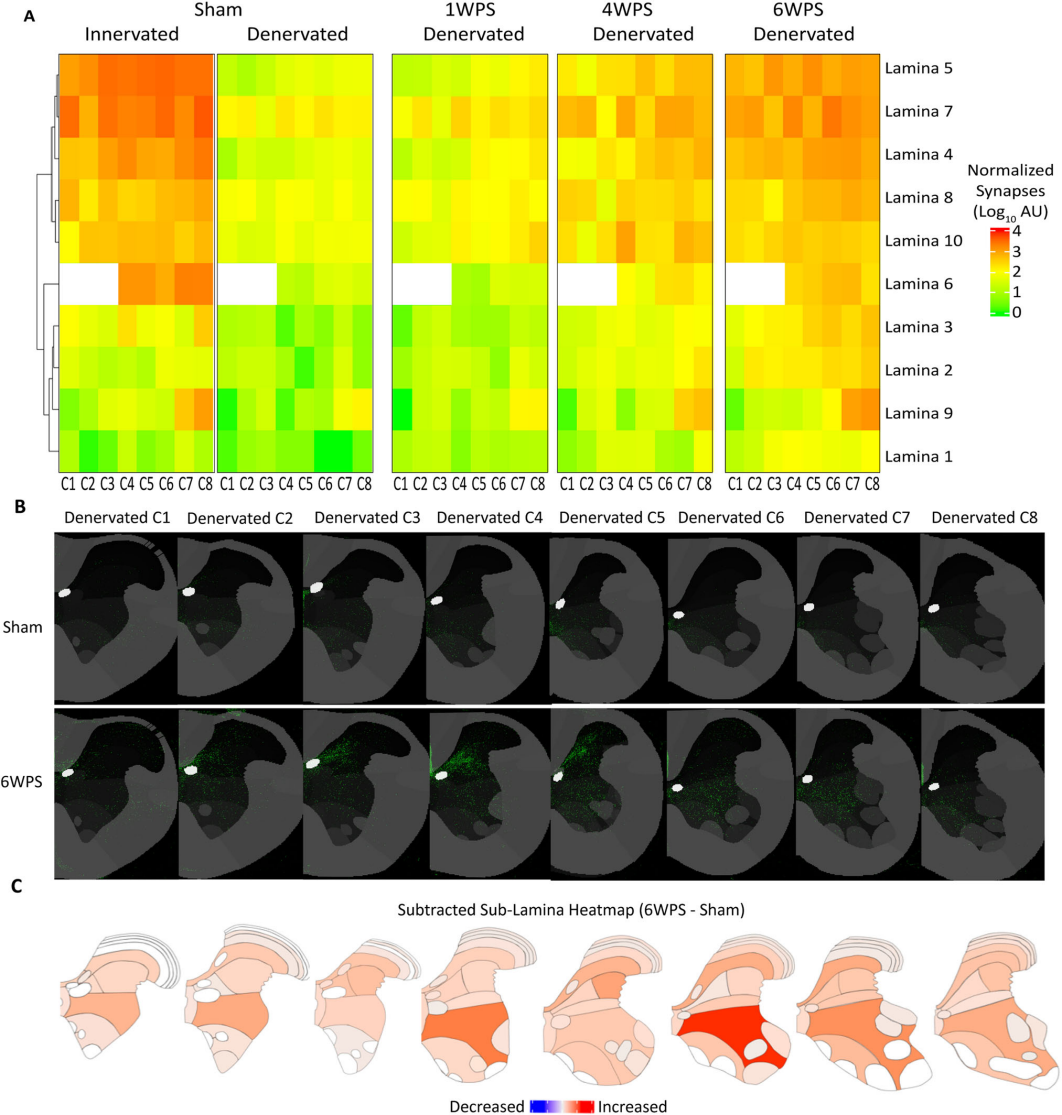
Level and lamina-specific reinnervation of the denervated hemicord by the contralesional CST synaptic projectome. ***A***, Heatmap demonstrating the mean of the group at each cervical level and lamina clustered using a dendrogram for the lamina of the sham innervated side. Only the denervated hemicord is shown for the three post-stroke groups. ***B***, Comparison of a sham and 6 week post-stroke animal with similar prenormalization viral labeling using condensed synapse probability maps across for each cervical level. ***C***, Heatmaps showing subtracted group mean of presynaptic terminals within atlas regions (6weeks post-stroke minus sham means) (Sham: *n* = 4, 1 WPS: *n* = 4, 4 WPS: *n* = 5, 6 WPS: *n* = 4).

One week post-stroke, no significant synaptic remodeling was measured, consistent with prior findings (Kaiser et al., 2019; Fig. 6*B*,*D*). By 4 weeks post-stroke, increased CST synaptic terminal signal was present in the denervated cord relative to sham conditions (log_10_FC = 1.56). Synapses were significantly enriched at levels C3 (log_10_FC = 1.45, *p* < 0.05) and C4 (log_10_FC = 2.45, *p* < 0.05; Fig. 6*D*) and laminae 5 (log_10_FC = 1.62) and 7 (log_10_FC = 1.27), mirroring CST innervation patterns in uninjured mice (Fig. 7*A*). Additionally, increased presynaptic terminals were observed in laminae 4, 6, 8, and 10, regions containing interneurons that modulate descending CST motor signals, with the most notably elevated synaptic terminals in lamina 10 at cervical level C4 (Fig. 7*A*; Sengul and Watson, 2012; Krotov et al., 2017; Gatto et al., 2021). At 6 weeks post-stroke, presynaptic terminals in the denervated hemicord remained elevated (log_10_FC = 1.82; Figs. 6*C*,*D*, 7*A*). The greatest CST synaptic signal was found in the most significantly denervated regions, C2, C4, C5, and lamina 7 (*p* < 0.05; Fig. 6*D*). We found robust changes in signal in lamina 7 at C4 and C6 (Fig. 7*A–C*). In addition, lamina 4, one of the smallest spinal laminae, received proportionally more signal, perhaps due to reinnervation of the internal basilar nucleus, a region known to integrate descending input from the sensorimotor cortex with ascending sensory information (Watson et al., 2021; Fig. 7*A*).

### Post-stroke CST reinnervation of the denervated lamina 9

**Figure 8.**
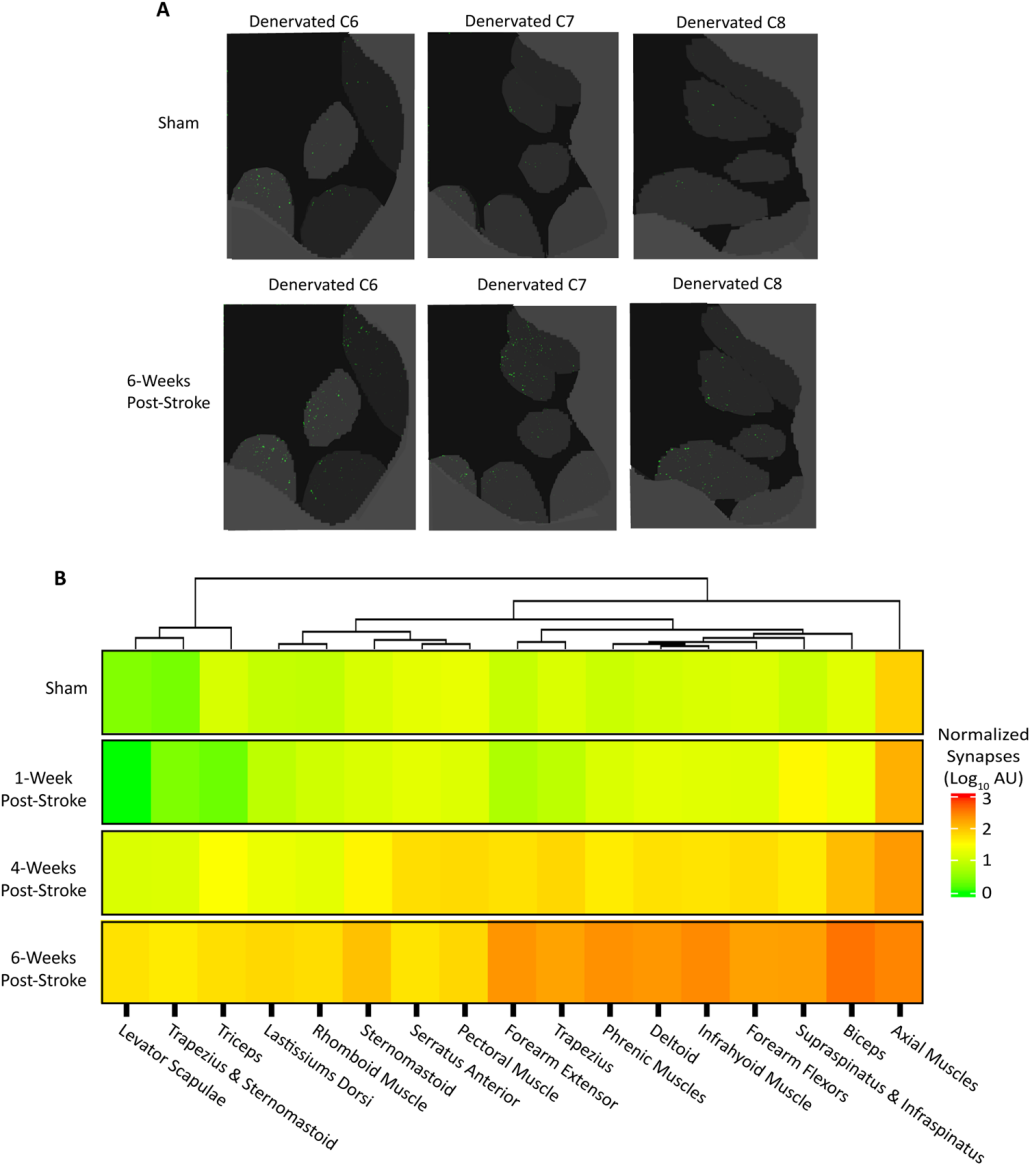
Increase in direct CST synaptic connections within specific laminae 9 motor pools after stroke. ***A***, Cervical levels C6–C8 from a sham and 6 week post-stroke animal with similar prenormalization viral labeling showing condensed presynaptic terminal probability maps for each cervical level. Only ventral motoneuron pools are shown for clarity. ***B***, Heatmap demonstrating the mean of the group at each lamina 9 motor pool of the denervated hemicord annotated in the atlas, clustered using a dendrogram for sham denervated (Sham: *n* = 4, 1 WPS 1: *n* = 4, 4 WPS: *n* = 5, 6 WPS: *n* = 4).

While some evidence supports direct CST sprouting onto spinal motoneurons after injury (Weidner et al., 2001), this has not yet been observed in mouse models. Beginning at 4 weeks post-stroke, we found that the uninjured CST significantly innervated lamina 9 in the denervated hemicord (log_10_FC = 1.56). Lamina 9, rather than lamina 7, received the greatest proportion of ipsilateral inputs in stroke animals (Fig. 7*A–C*). These ipsilateral connections were localized to multiple motoneuron pools, including the forearm flexors (log_10_FC = 1.8) and axial musculature (log_10_FC = 1.22) a clear deviation from normal CST anatomy (Fig. 8*B*). By 6 weeks post-stroke, there was a notable increase in nearly all ipsilateral motoneuron pools relative to sham (log_10_FC = 1.98), particularly in cervical levels with the most spinal motor neurons in C7 and C8 (Fig. 8*A*). Along with a visually appreciable increase in presynaptic terminals within motoneuron pools, these findings suggest that lamina 9 is a prominent target for the uninjured CST after stroke (Fig. 8*A*).

## Discussion

This paper describes the development of an automated 3D microscopy and image analysis pipeline, SpinalTRAQ, which provides quantitative feature assessment of the mouse cervical spinal cord at micron resolution. As part of this pipeline, we established the SpinalTRAQ Common Reference Atlas (ST-CRA), the first high-resolution, volumetric template that captures the true complexity and variability of cervical spinal cord architecture. This enabled precise, lamina- and level-specific quantification of the bilateral CST cervical spinal synaptic projectome in health and after stroke. Under healthy conditions, we confirmed that the CST predominantly targets laminae 5 and 7 (Fiederling et al., 2021a, 2021b). We also observed previously unknown targets of the ipsilateral CST, including lamina 7 and, to a lesser extent, laminae 8, 10, and 9. We additionally established that the ipsilateral CST does not project to laminae 4 or 5, regions that receive comparatively more synaptic input in the contralateral hemicord. Our findings reveal two distinct patterns of remodeling in the ipsilateral hemicord after motor cortex stroke injury: (1) homotopic reinnervation of premotor laminae and (2) the emergence of novel corticomotoneuronal connections within lamina 9 of caudal cervical regions. Furthermore, the lamina 9 plasticity is robust and highly localized to C7 and C8 forearm flexor and extensor motoneuron pools, aligning with the specific motor impairments produced by our photothrombotic stroke model (Becker et al., 2016, 2020). Overall, these findings highlight the use of unbiased, quantitative measures of neuronal circuit remodeling, having identified novel features of CST anatomy and post-stroke plasticity.

SpinalTRAQ provides several advantages that enable the identification of previously unrecognized CST targets. Specifically, STPT utilizes block-face imaging of whole tissues that overcomes many limitations of conventional microscopy approaches, including sampling bias, tissue distortion, and time-intensive manual processing (Liu et al., 2013). Like whole-brain analyses, surveying the entire cervical spinal cord achieves a level of coverage that has been proven effective for uncovering novel post-injury remodeling (Bachmann et al., 2014; Poinsatte et al., 2019; Wang et al., 2022). Combined with a rostrocaudal sampling frequency of 20 µm and quantitative measurement of both presynaptic terminals and axonal projections, we achieved a drastically more comprehensive assessment of the CST compared with prior mapping studies. For example, we detected sparse ventral and dorsolateral CST labeling, contrary to reports that these pathways are eliminated by postnatal day 14 (Gu et al., 2017). Such rare projections would likely be missed without the 100× increase in sections per animal, aligning with previous findings that these pathways are highly sensitive to the visualization technique (Steward et al., 2004; Bareyre et al., 2005; Steward et al., 2008; Hilton et al., 2016). Likewise, using a conventional histological approach of quantifying three cervical sections per animal would greatly limit the chances of detecting lamina 9 projections in either healthy or post-stroke conditions.

STPT pipelines are well-established tools for whole-brain assessments in healthy and pathological states, including stroke (Poinsatte et al., 2019; Ramirez et al., 2019; Lu et al., 2021; Liwang et al., 2023; Ramirez et al., 2023; Watakabe et al., 2023; Watakabe et al., 2025). To develop the first STPT workflow adapted for the cervical spinal cord, we created the ST-CRA, which builds on the previously established atlas SpinalJ, the first 3D microscopy reference atlas for the mouse spinal cord. Noted limitations of this reference atlas (Fiederling et al., 2021a,b) include its basis on eight cervical spinal cord sections from a single reference animal. To address this, we generated a high-resolution average template built from 620 sections per mouse across 20 animals. By incorporating SpinalJ atlas annotations into this average template, we created the ST-CRA, which serves as an improved reference atlas for capturing anatomical variations along single cervical segments and between animals. The ST-CRA has several limitations. First, we consistently observed GFP+ synaptic signals that appear to be present in white matter tracts (Fig. 3*F*, Extended Data Fig. 3-1*A*). Some of these “white matter synapses” surround the gray/white matter intersection, a highly variable boundary that presents a significant challenge for precise registration. While some of these synapses might represent erroneous location assignments, the most common explanation is that the gray–white boundary is not regular: neurons from laminae 4 to 9, known CST targets, commonly extend dendritic projections deep into white matter regions such as the ventrolateral funiculus (Extended Data Fig. 3-1*A*). These individual projections would not appear on the average image. Nevertheless, more accurate annotations throughout the spinal cord can be achieved through deep learning-based segmentation models that more readily factor variable anatomical features. While this technology is not currently developed, future reference atlases should rely on these methods once they become available (Iqbal et al., 2019a,b). Second, volumetric imaging methods, such as light sheet, STPT, or confocal imaging, greatly benefit from 3D-to-3D registration afforded by the ST-CRA. Although we present the methodology for registering STPT images, modified workflows can be created which allow registration of confocal or light sheet volumes. This can be achieved using cross-modality registration or via creation of additional average template volumes, which will be generated in future work (Chandrashekhar et al., 2021). This would be especially advantageous for imaging samples requiring immunofluorescence, since STPT is generally limited to capturing endogenous fluorescent signal. Third, the ST-CRA can only be used to register cervical spinal cord images. We initially focused on this region because of its key role in driving fine motor recovery, addressing one of the most common long-term deficits after stroke. While outside the scope of this study, future efforts will support the development of a complete reference atlas spanning the entire rostrocaudal extent of the mouse spinal cord.

Precise quantification of whole cervical cord volumes revealed novel findings for healthy CST anatomy, particularly differences in ipsilateral versus contralateral connectivity that may have important implications for motor function. Consistent targeting of ipsilateral CST projections to laminae 7, 8, and 10 and axial motoneuron pools suggests a potential role in motor control that warrants further investigation. These spinal laminae are known to be functionally distinct targets of the contralateral CST: laminae 7 and 8 receive input primarily from the contralateral rostral forelimb area, while lamina 10 is targeted by projections from the contralateral caudal forelimb area (Carmona et al., 2024). Whether ipsilateral CST connections to these laminae preserve this behaviorally relevant pattern of connectivity remains unknown. This question is especially important in the context of stroke. In smaller cortical lesions, peri-infarct tissue harbors spared CST connections that can retain control of the impaired limb. However, in larger strokes, the intact hemisphere may provide optimal control of motor function, particularly the ipsilateral CST fibers, which are anatomically positioned to reinnervate interneurons and motoneurons that have lost input.

After stroke, homotopic reinnervation of ipsilateral laminae 4, 5, and 7 by the intact CST may represent restored connectivity to key interneuronal populations (Ueno et al., 2018), including V2a neurons (Bui et al., 2013; Levine et al., 2014; Hayashi et al., 2018; Kaiser et al., 2019), motor synergy encoders (Kullander et al., 2001; Restrepo et al., 2011; Paixao et al., 2013; Chedotal, 2019), dl3 premotor interneurons (Kuang and Kalil, 1990; Weidner et al., 2001; D'Acunzo et al., 2014; Fait et al., 2024), and V1 interneurons (Chapman et al., 2025), which are essential for motor coordination and recovery after injury (Heffner and Masterton, 1975; Bortoff and Strick, 1993). The spatial targeting of CST terminals supports the idea that spinal synaptic remodeling is tightly regulated, in a similar manner to supraspinal connections (Weidner et al., 2001; Poinsatte et al., 2019; Wang et al., 2022). This aligns with prior findings that CST sprouting in laminae 6–9 promotes motor recovery after stroke, whereas sprouting in dorsal laminae does not (Armand et al., 1997; Olivier et al., 1997). Notably, the current studies were conducted exclusively in male mice and thus may miss sex-dependent findings. Current studies of sex differences in axonal sprouting or regeneration in the CNS are extremely limited (Loy and Milner, 1980; Milner and Loy, 1982; Jang et al., 2021), and future efforts to generate a sex-specific reference atlas and ST-CRA would greatly benefit translational investigations of CST spinal anatomy in health and disease.

Direct CST–motoneuron connectivity is thought to underpin the increased manual dexterity and strength of primates (Lemon, 2008; Lemon, 2019) and in developing mice (Gu et al., 2017). In rodents, data are limited and debated (Yang and Lemon, 2003; Alstermark et al., 2004) despite long-standing evidence for these connections (Weidner et al., 2001; Bareyre et al., 2005; D'Acunzo et al., 2014; Maeda et al., 2016; Ueno et al., 2018; Fageiry et al., 2024; Fait et al., 2024). In our studies, monosynaptic tracing revealed CST contacts on lamina9 motoneurons in both healthy mice and after stroke. In fact, with evidence that spinal motoneuron dendrites often extend into laminae 7 and 8 (Fig. 1*E*), it is possible that our quantification of CST–motoneuron contacts was underestimated. Corticomotoneuronal connections were identified using an AAV1 viral vector expressing Cre in nuclear GFP reporter mice. This monosynaptic labeling method requires active synaptic release (Zingg et al., 2020) and is well validated in numerous studies, including prior observations of direct CST–motoneuron targeting (Zhao et al., 2017; Kumamaru et al., 2019; Umaba et al., 2021; Kathe et al., 2022; Kitanishi et al., 2022; Atsumi et al., 2023; Fait et al., 2024; Fan et al., 2024; Karube et al., 2024; Narai et al., 2024; Fujiyama and Karube, 2025; Nimitvilai-Roberts et al., 2025). When delivered in Cre-dependent reporter mice, AAV1-Cre demonstrates highly restricted anterograde spread to first-order neuronal connections from glutamatergic and GABAergic neurons, more limited retrograde monosynaptic spread, and minimal toxicity of transfected cells. The AAV1-cre system has been reported to label some non-neuronal targets (oligodendrocytes and astrocytes), possibly due to viral leakage or phagocytosis of Cre-containing degenerating axons or synapses (Wahl et al., 2014). While previous studies have shown CST extension into the ventral column after spinal cord injury (Hayashi et al., 2018) and direct CST–corticomotoneuronal connections after caudal CST lesions (Weidner et al., 2001), this study is the first to demonstrate its emergence following motor cortical stroke. These connections appear sparse but may represent latent circuits recruitable under specific conditions, such as injury or altered motor demands. Further investigation should clarify whether they contribute to dexterous motor output or recovery following cortical or spinal injury.

In summary, this study uses the novel platform SpinalTRAQ to provide new insights into CST synaptic organization and plasticity, highlighting striking organization of ipsilateral CST projections in healthy animals and homotopic reinnervation of premotor laminae and novel corticomotoneuronal connectivity after stroke. SpinalTRAQ represents a versatile tool for spinal circuit analysis, offering unprecedented spatial resolution to explore synaptic changes in health, development, and disease. Future research using this pipeline in the context of stroke should focus on three key areas. First, the unique ipsilateral CST innervation observed in healthy mice warrants further exploration to determine its role in motor control and its potential for aiding recovery after injury. Second, longitudinal studies are needed to correlate CST remodeling with behavioral recovery, clarifying the functional roles of homotopic and corticomotoneuronal plasticity. Ultimately, understanding the molecular mechanisms that govern CST reinnervation will facilitate the identification of targets to enhance adaptive plasticity while minimizing maladaptive changes.

## Synthesis

Reviewing Editor: Gergely Silasi, University of Ottawa Decisions are customarily a result of the Reviewing Editor and the peer reviewers coming together and discussing their recommendations until a consensus is reached. When revisions are invited, a fact-based synthesis statement explaining their decision and outlining what is needed to prepare a revision will be listed below. The following reviewer(s) agreed to reveal their identity: NONE. Note: If this manuscript was transferred from JNeurosci and a decision was made to accept the manuscript without peer review, a brief statement to this effect will instead be what is listed below.

The authors have clearly addressed the concerns raised by previous reviewers, and the manuscript describes a novel tool for localizing synaptic contacts in the spinal cord. The application of the tool following stroke injury also provides further validity for the approach. It may be beneficial to state in the introduction or discussion that after small cortical lesions peri-infarct tissue may control the impaired limb, whereas after large lesions the intact hemisphere may provide optimal control the impaired limb.
