# Supplementary figures and images for: SpinalTRAQ: A Novel Pipeline for Volumetric Cervical Spinal Cord Analysis Identifies the Corticospinal Tract Synaptic Projectome in Healthy and Post-stroke Mice
Source: eNeuro. 2025 Sep 18;12(9):ENEURO.0276-25.2025. doi: 10.1523/ENEURO.0276-25.2025 (PMC12456858; doi:10.1523/ENEURO.0276-25.2025)

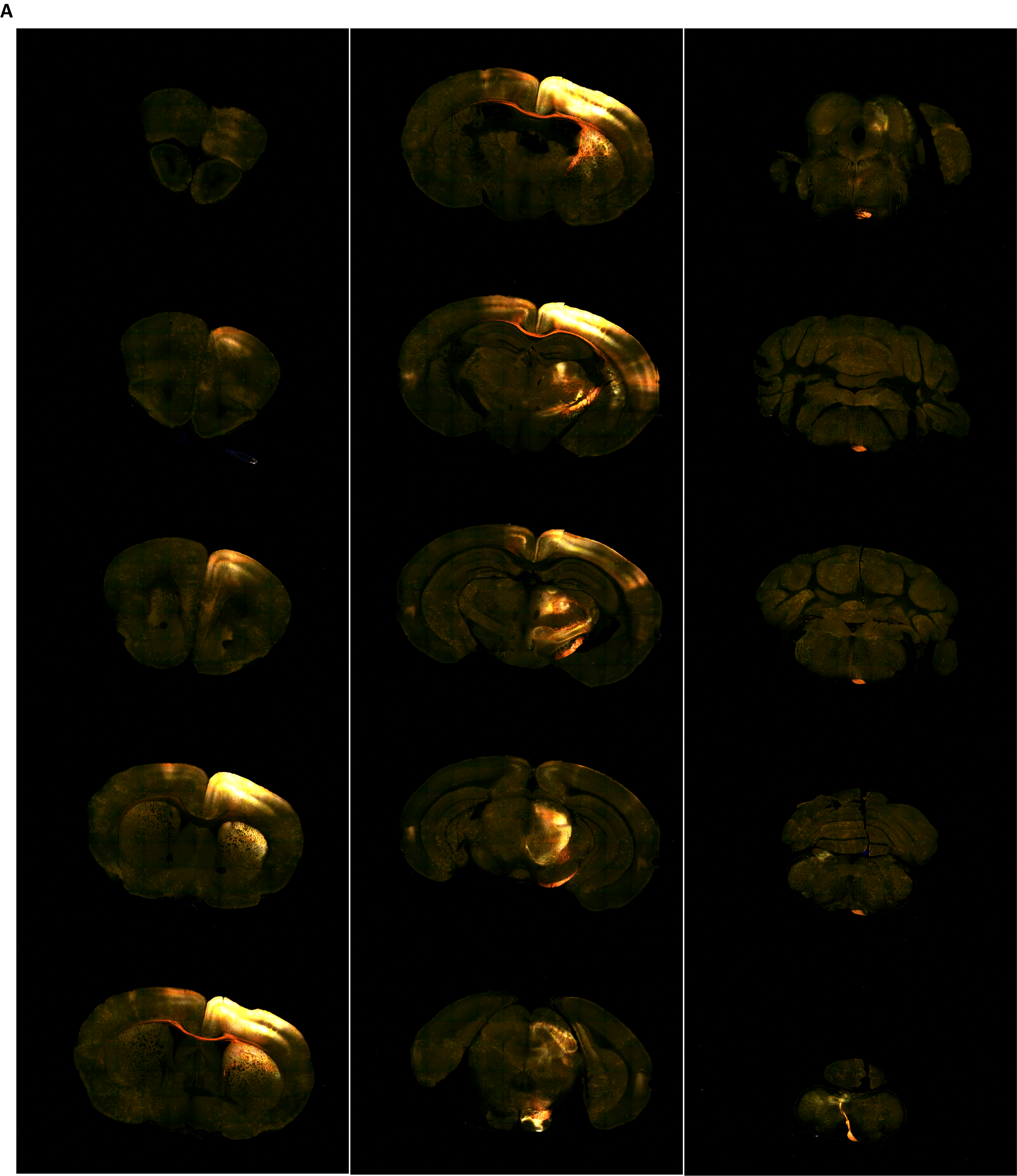

Supplement: Figure 2-1 — Whole brain SynaptoTag4 STPT imaging (A) STPT subset of whole brain imaging of SynaptoTag4 inject healthy adult mouse with images spaced 975μm apart from each subsequent image. Download Figure 2-1, TIF file. [file eneuro-12-ENEURO.0276-25.2025-s001.tif]

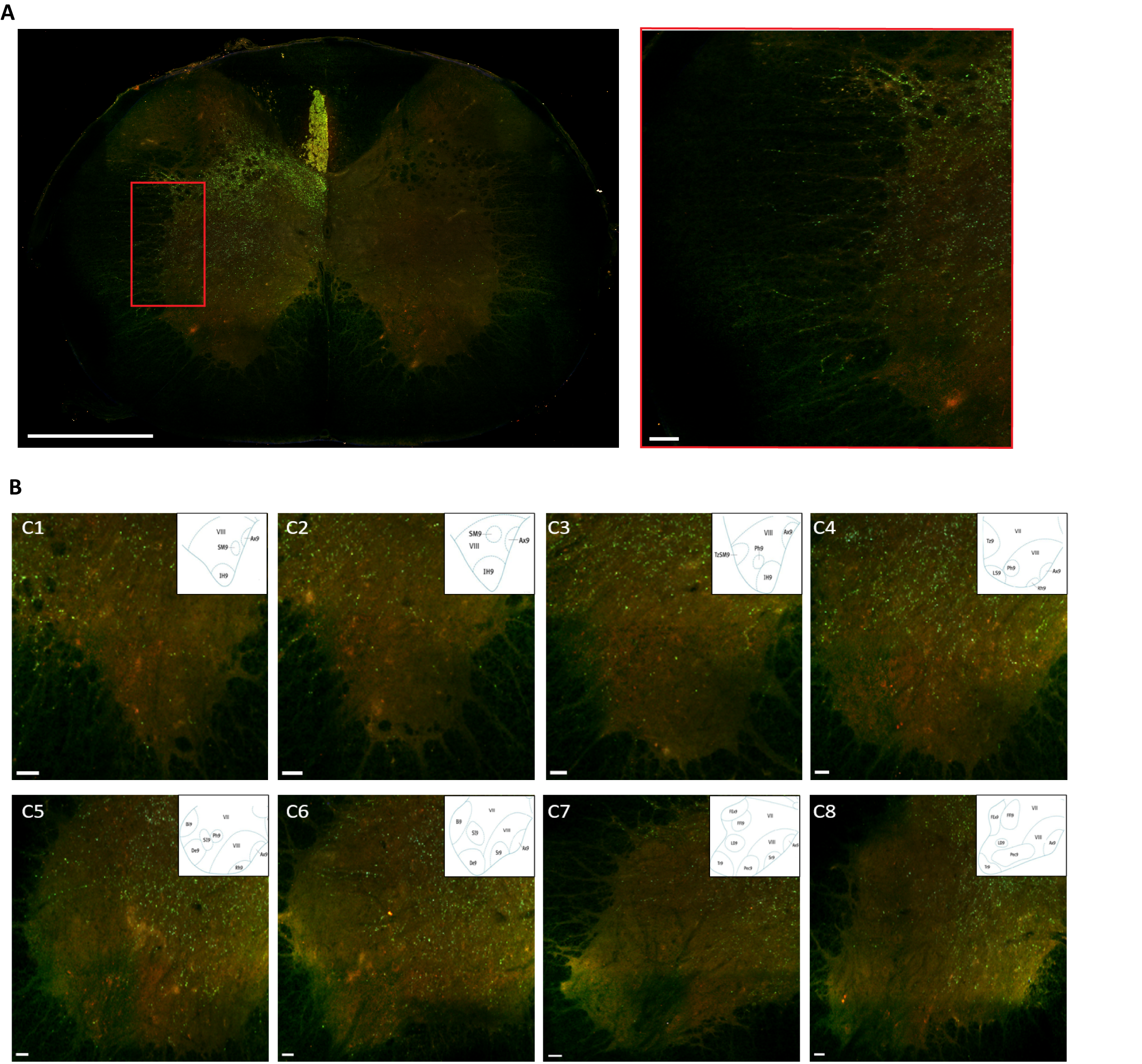

Supplement: Figure 3-1 — CST Synapses in traditional white matter regions and ventral horn. (A) (Left) eGFP + presynaptic terminals in raw fluorescent image of a healthy adult mouse spinal cord section (scale bar 400μm). (Right) Zoomed inset of left image of eGFP + presynaptic terminals in gray-matter dendritic arbors into lateral funiculus (scale bar 50μm). (B) eGFP + presynaptic terminals in zoomed ventral horn raw fluorescent image of a healthy adult mouse spinal cord section from C1-C8 with Allen Spinal Cord Atlas lamina 9 inset (scale bar 50μm). Download Figure 3-1, TIF file. [file eneuro-12-ENEURO.0276-25.2025-s002.tif]
